# Long-term Death Rates, West Nile Virus Epidemic, Israel, 2000

**DOI:** 10.3201/eid1111.040941

**Published:** 2005-11

**Authors:** Manfred S. Green, Miriam Weinberger, Judith Ben-Ezer, Hanna Bin, Ella Mendelson, Dan Gandacu, Zalman Kaufman, Rita Dichtiar, Annette Sobel, Dani Cohen, Michal Y. Chowers

**Affiliations:** * Israel Center for Disease Control, Tel Hashomer, Israel; † Tel Aviv University, Tel Aviv, Israel; ‡ Rabin Medical Center, Petach Tikva, Israel; § Central Virology Laboratory, Tel Hashomer, Israel; ¶ Ministry of Health, Jerusalem, Israel; # State of New Mexico Office of Homeland Security, Santa Fe, New Mexico, USA; ** Meir Medical Center, Kfar Sava, Israel

**Keywords:** West Nile virus, mortality, follow-up, prognostic factors, dispatch

## Abstract

We studied the 2-year death rate of 246 adults discharged from hospital after experiencing acute West Nile Virus infection in Israel during 2000. The age- and sex-adjusted death rates were significantly higher than in the general population. This excess was greater for men. Significant adverse prognostic factors were age, male sex, diabetes mellitus, and dementia.

West Nile virus (WNV) was first isolated in 1937 (*1**,**2*) and has since been found in Europe, Africa, Asia, and North America (*1*). Human WNV infection was first documented in Israel in 1951 (*3*). Several outbreaks occurred during the 1950s and during the following 40 years, but mainly sporadic cases occurred (*4*). In 2000, an epidemic of 428 serologically confirmed cases led to 42 deaths during the acute phase of the illness (*5*). Little has been reported in the literature on the long-term sequelae of WNV infection. In 1953, 70 patients 18–20 years of age were followed up for <11 months, and no serious sequelae or deaths were observed (*6*). Although 12% of the patients in the New York outbreak in 1999 died during the acute phase of the illness, no deaths occurred among the 35 patients followed up for 1 year after hospitalization (*7*). The aim of the present study was to determine whether infection with WNV has long-term effects on death rates and to evaluate variables such as age, sex, symptoms and signs, and coexisting conditions as potential prognostic factors.

## The Study

Between July 1 and November 30, 2000, 428 patients with serologic diagnoses of WNV infection were reported to the Ministry of Health (MOH) Department of Epidemiology. Diagnosis of WNV infection was made on the basis of symptoms, signs, and laboratory confirmation of the presence of immunoglobulin M (IgM) antibodies. All serologic tests were performed in the MOH's Central Virology Laboratory by using an IgM-capture enzyme-linked immunosorbent assay in serum or cerebrospinal fluid samples of patients (*8*). At the time of the epidemic, this was the working definition of a case of WNV infection. While WNV IgM antibodies can persist >1 year (*9*), a seroepidemiologic study in a healthy population in Israel at the time of the outbreak, using the same laboratory methods, found only 1.2% had IgM antibodies (M.Y. Chowers et al., unpub. data). Thus, few, if any, false-positive results would be expected.

The study population was limited to the 326 hospitalized patients. After excluding 34 patients <20 years of age and the 46 patients who died during hospitalization or within 1 week of discharge, 246 survivors were eligible for follow-up. Demographic data on the cases and date of onset of the disease were obtained from the Department of Epidemiology. The mean ages of the patients were 57.8 years for men and 62.8 years for women. Clinical data on symptoms and coexisting conditions were obtained from the hospital discharge letters. Of eligible survivors, 48.3% had diagnoses of encephalitis or meningoencephalitis, 13.5% had a diagnosis of meningitis, 28.2% had fever, rash, or both, and the other 10% of the survivors had other clinical symptoms.

The patients were monitored to determine whether any deaths occurred from date of hospital discharge until November 30, 2002. Deaths were determined by matching the identity numbers of the case-patients with the data in the national population registry maintained by the Ministry of Interior. We were able to confirm all deaths in the cohort during this period. By November 30, 2002, after an average follow-up period of 24 months (median 26 months, range 1–29 months), 30 of 246 patients had died.

Death certificates were obtained for all case-patients, and all causes of death were recorded and coded by using the International Classification of Disease, Ninth Revision. The coding was reviewed independently by another coder, and the underlying cause of death was determined by using World Health Organization criteria.

Bivariate comparisons between characteristics of the patients who died and the survivors were carried out by using the χ^2^ and *t* tests. Kaplan-Meier survival curves were constructed for all patients and for men and women separately, and the difference between men and women was compared by using the log-rank test. The age-standardized mortality ratios (SMRs) were computed by using the person-time method with the PAMCOMP program (Institute of Epidemiology and Social Medicine, University of Muenster, Muenster, Germany) (*10*). SMRs were calculated by dividing the observed number of deaths by the expected number. Person-months of risk were calculated from the reported onset of the disease until either death or November, 30, 2002, whichever occurred first. The expected number of deaths in the study cohort was calculated by multiplying person-months at risk by the national death rates in 1997 (the last year for which detailed published data are available) for the same age and sex categories. Exact 95% confidence intervals (CIs) and p values were calculated by using the Poisson distribution. Attributable risk was computed by the formula (SMR-1)/SMR. Prognostic factors were evaluated by using Cox proportional hazards regression analysis to estimate adjusted rate ratios while adjusting for other potential determinants of death. The hazard rate ratio (RR) is defined as the ratio of the mortality rate in the group of patients with a given study factor to the rate in those without the factor. We calculated 95% CIs for each adjusted RR. The SAS package version 8.2 (SAS, Cary, NC, USA) was used for all calculations other than the SMRs.

**Figure 1:**
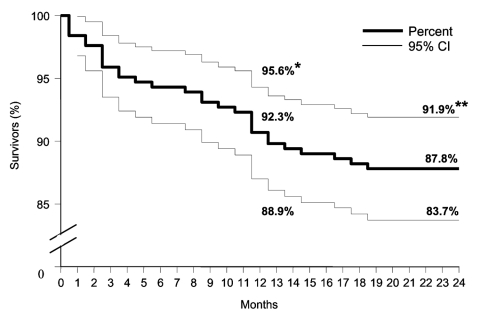
Kaplan-Meier survival curves for 2-year mortality follow-up of 246 patients discharged from hospital after West Nile virus infection during the epidemic in Israel in 2000. *Survival after 1 year; **survival after 2 years; CI, confidence interval.

**Figure 2:**
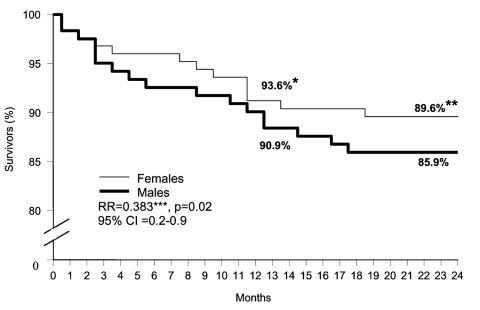
Kaplan-Meier survival curves for 2-year mortality follow-up of 246 patients discharged from hospital after West Nile Virus infection during the epidemic in Israel in 2000, by sex. *Survival after 1 year; **survival after 2 years; ***relative risk (RR) for women compared with men, adjusted for age, diabetes, ischemic heart disease, immunodeficiency, cerebrovascular disease, hypertension, and dementia.

The Kaplan-Meier survival curves are shown for the total cohort and for men and women separately (Figures 1 and 2). Overall, after 1 year, 7.7% had died and after 2 years, 12.2%. After 1 year the death rate was 6.4% for women compared with 9.1% for men (p = 0.025), and after 2 years it was 10.4% for women and 14.1% for men (p = 0. 021).

Age-adjusted SMRs, both sex-specific and sex-adjusted, at 6 months, 1 year, and the end of the follow-up, are shown in Table 1. The overall SMR at 1 year was nearly 2.5 times higher than expected (SMR = 2.49, 95% CI 1.50–3.89). Among men, the death rate was >3 times higher than expected, whereas among women the death rate was less than twice as high and not significant. The excess risk at the end of the second year was lower and not significant. Overall, in 50% of the deaths the underlying cause was ascribed to cardiovascular disease. In only 20% was WNV infection given as the underlying cause of death, although this result is clearly influenced by coding practices.

**Table 1:** Standardized mortality ratios (SMRs) for 246 patients >20 years of age discharged from hospital after West Nile virus infection, Israel, 2000, compared with those for the general population during 2 years of follow-up*

Follow-up period	No.	Observed deaths	Expected deaths	SMR	95% CI
First 12 mo					
Men	121	11	3.47	3.17†	1.58–5.67
Women	125	8	4.16	1.92†	0.83–3.79
Total	246	19	7.63	2.49‡	1.50–3.89
12–24 mo					
Men	110	6	2.91	2.06†	0.75–4.49
Women	117	5	3.74	1.34†	0.43–3.12
Total	227	11	6.64	1.66‡	0.83–2.96

*CI, confidence interval. †Adjusted for age. ‡Adjusted for age and sex.

In the Cox regression analyses for evaluating demographic factors and the symptoms and signs as prognostic factors, while simultaneously controlling for each, only age, and especially the oldest age group tested (>85 years), was a significant adverse prognostic factor (data not shown). Possible interactions between the symptoms and signs with age and sex were not significant. A second Cox regression analysis for evaluating coexisting conditions as prognostic factors is shown in Table 2. After age, sex, and the other coexisting conditions were controlled for, only diabetes mellitus and dementia remained as significant, independent predictors of death (RR = 2.74 and 2.94 for diabetes mellitus and dementia, respectively). Again, interaction effects were not significant.

**Table 2:** Cox proportional hazards analysis for the association between coexisting conditions and 2-year death rate follow-up of 246 patients >20 years discharged from hospital during a West Nile virus epidemic, Israel, 2000*

Variable	B	RR	95% CI	p
Age† (y)				
75–84	2.00	7.38	2.77–17.71	<0.0001
>85	2.49	12.10	4.24–34.49	<0.0001
Sex (M = 0, F = 1)	–1.09	0.33	0.14–0.79	0.0124
Diabetes (no = 0, yes = 1)	1.01	2.74	1.11–6.81	0.0294
Ischemic heart disease (no = 0, yes = 1)	0.17	1.19	0.52–2.72	0.6794
Immunodeficiency (no = 0, yes = 1)	0.04	1.04	0.13–8.08	0.9696
Cerebrovascular disease (no = 0, yes = 1)	–1.22	0.29	0.07–1.28	0.1023
Hypertension (no = 0, yes = 1)	0.09	1.09	0.48–2.48	0.8365
Dementia (no = 0, yes = 1)	1.08	2.94	1.09–7.91	0.0330

*B, natural logarithm of the hazard rate ratio; RR, hazard rate ratio; CI, confidence interval. †The age group <75 years old was set as a reference.

## Conclusions

In this study, excess deaths occurred among survivors compared with the general population. This excess appeared to occur mainly in the first year after the acute illness and was more prominent among men than women. Older age, and especially the oldest age group tested (>85 years), diabetes mellitus, and dementia were other significant independent predictors of death.

To demonstrate the magnitude of this excess risk, we compared these findings with an Israeli study of deaths within 1 year after hospital discharge among patients with acute myocardial infarction of whom ≈30% had diabetes (S. Bachar, Acute Coronary Syndrome in Israel 2000 study, unpub. data). After age and sex were controlled for, the death rate within 1 year for WNV-infected patients in the present study was similar to that of patients after myocardial infarction (7.7% vs. 9.5%, p = 0.881 ). This finding indicates that the 1-year postdischarge death risk in the WNV-infected patients is of a similar magnitude to that of patients with a severe, acute, noninfectious illness.

The main negative prognostic factors in the present study were older age, male sex, diabetes mellitus, and dementia. Similar associations with deaths in the acute phase of the illness have been observed for age (*5**,**11*) and diabetes mellitus (*5*). Thus older age and possibly diabetes mellitus influence both acute-phase and long-term mortality rates. Why long-term mortality rates, not related to preexisting illnesses or age, are higher in men than in women is unknown. In several studies of clinical WNV infection, men appeared to have a higher incidence of disease, but this difference has not been observed in Israel (*5*).

Survivors of herpes simplex encephalitis have a shorter life expectancy (*12**,**13*), and to a lesser extent, so have patients with other viral encephalitides (*13*). Some of these excess deaths could be attributed to severe neurologic impairment and instability, while other deaths were unrelated to the infection.

The reasons for the increased long-term death rate in survivors of acute WNV infection are not clear. The clinical features described in WNV encephalitis have been described as typical for arboviral encephalitides (*1*). Severe neurologic sequelae have been described in survivors of invasive WNV infection, and a substantial number of patients do not regain their baseline function (*8*). This deficit could contribute to an increase in deaths.

In this epidemic in Israel, 2 clades of WNV were isolated (*8**,**14*). One was closely related to the 1999 New York isolate and the other to a 1999 Russian isolate and a 1997 Romanian isolate (*14*). The outbreak was characterized by relatively high case-fatality rates among hospitalized patients; 14% of the patients died during the acute phase (*5*). This rate is similar to that observed in the New York outbreak (*15*). No evidence suggests that the virus in Israel was any different in virulence.

The results of this study on the potential long-term impact of WNV infection are relevant for hospitalized patients. They may have been more susceptible to clinical WNV infection. The significant excess death rate indicates that the combined case-fatality rate in all hospitalized patients in the acute and convalescent phases could be >10%, and in patients >65, >30% (*5*). Thus, such patients require long-term monitoring and support. Possible sex differences in death rates require further investigation.
